# 2191: The SDM learning loop model

**DOI:** 10.1017/cts.2017.253

**Published:** 2018-05-10

**Authors:** Sarah Ronis, Kurt Stange, Lawrence Kleinman

**Affiliations:** Case Western Reserve University, Cleveland, OH, USA

## Abstract

OBJECTIVES/SPECIFIC AIMS: (1) To propose an iterative decision-making model of care planning for CSHCN. (2) To identify targets warranting measurement in future studies of SDM in care planning for CSHCN. METHODS/STUDY POPULATION: Conceptual model developed by a multidisciplinary team iteratively considering the complex relationships among diverse factors affecting care planning for CSHCN, informed by clinical and implementation science experience and a scoping literature review of medical and cognitive sciences literature addressing interpersonal decision-making, communication, negotiation, and trust among children, their parents, and their clinicians. RESULTS/ANTICIPATED RESULTS: Decision-making interventions in pediatrics tend to focus narrowly on single acute decisions, providing minimal guidance for decisions related to chronic disease management over time. Few models account for the role of the child in the decision-making process, despite their ongoing development. Therefore, we propose a model of shared decision-making in the context of managing chronic illness in children that recognizes all actors and can support both the design of clinical care and research. This model—The SDM Learning Loop Model—highlights the dynamic iterative nature of exchanges between and among the clinical team and the parent-child dyad and recognizes the child as the center of each decision-making cycle. The model accounts for key practice, family, experiential, and emotional contexts influencing the decision-making encounter. In this model, change in child health status and developmental capacity resulting from a given cycle’s care plan will directly influence the relationship between clinician and parent-child dyad (eg, mutual trust, attunement) and impact each party’s engagement in the next round of decision-making. The relationship between experience and outcome stimulates learning. DISCUSSION/SIGNIFICANCE OF IMPACT: Our proposed SDM Learning Loop Model suggests that increasing the shared nature of decision making is not only likely to optimize care planning, but creates “buy-in” that can both reinforce the impact of positive outcomes, and moderate the negative impact on relationships when the outcome is other than desired. We hypothesize that this model can guide care planning and shape research to the benefit of both clinical outcomes and clinician-family relationships. Future work should focus on the development and validation of measures to account for the experiential and emotional contexts in which such decisions are made, and the outcomes of care in this population.

